# Prospective randomized comparison of the safety, efficacy, and cosmetic outcome associated with mini-transverse and mini-longitudinal radical prostatectomy incisions

**DOI:** 10.4103/0970-1591.70563

**Published:** 2010

**Authors:** Bruce R. Kava, Rajinikanth Ayyathurai, Cynthia T. Soloway, Miguel Suarez, Prashanth Kanagarajah, Manoharan Murugesan

**Affiliations:** Department of Urology, University of Miami, Miami, Florida; 1 Department of Pathology, Veterans Affairs Medical Center, Miami, Florida

**Keywords:** Cosmesis, localized prostate cancer, prostate cancer treatment, radical prostatectomy

## Abstract

**Aims:**

Open radical retropubic prostatectomy (ORP) has traditionally been performed through a lower midline incision. Prior efforts to reduce pain and expedite recovery include a variety of alterations in length and the orientation of the incision. The aim of our study is to compare the safety, efficacy, and cosmetic outcomes associated with transverse and longitudinal mini-radical prostatectomy incisions.

**Materials and Methods:**

Consecutive patients undergoing ORP at a single institution were studied. Patients were randomized to receive either a modified transverse or longitudinal incision. In all patients, the length of the incision was 7cm. The following parameters were compared between the two groups: Perioperative blood loss, duration of surgery, technical factors, pain and analgesic requirements, length of hospital stay (LOS), and pathological stage. The Patient and Observer Scar Assessment Scale (POSAS) was used to compare the cosmetic aspects associated with the incisions.

**Results:**

Fifty-six patients underwent a transverse (n=27) and longitudinal (n=29) mini- incision ORP. No significant differences were noted in the perioperative parameters that were compared (*P*>0.116). None of the patients required blood transfusion, there were no wound complications. Perioperative pain and analgesic requirements were not significantly different among the two study arms (*P*>0.433). The POSAS indicated no significant difference in cosmesis scores with both incisions (*P*>0.09).

**Conclusions:**

Seven-centimeter transverse and longitudinal mini-incisions offer alternatives to the standard ORP incision, and to minimally invasive approaches. Both incisions are safe, associated with little postoperative pain, and a short postoperative LOS. Both incisions provide highly satisfactory cosmesis for the patient.

## INTRODUCTION

Open radical retropubic prostatectomy (ORP) has traditionally been performed through a lower midline, extraperitoneal incision.[1] Efforts to reduce postoperative pain and expedite recovery following prostate cancer surgery have focused on techniques that limit the length of the incision.[2,3] Other reports have altered the orientation and technique of the incision to achieve better outcomes.[4–6]

The purpose of this randomized study was to prospectively compare the safety, efficacy, and cosmetic outcomes associated with a modified transverse and a longitudinal mini-incision for ORP. Specific variables assessed included: estimated blood loss (EBL), duration of surgery, the ability of the incision to provide adequate exposure, perioperative pain and analgesic requirements, and postoperative length of the hospital stay (LOS). Unique to our study, we also evaluated the cosmetic aspects of these incisions as perceived by both the patient and the surgeon.

## MATERIALS AND METHODS

The study was approved by the Institutional Review Board. Consecutive patients undergoing ORP for clinically localized prostate cancer were eligible for participation. Following informed consent, patients were randomized to undergo either a transverse or longitudinal mini-incision ORP. A single surgeon performed the surgery for patients in both groups. Except for the orientation of the incision, all the other technical components of the surgery were left to the surgeon’s discretion. Demographic information was prospectively recorded prior to surgery.

**Figure 1:**
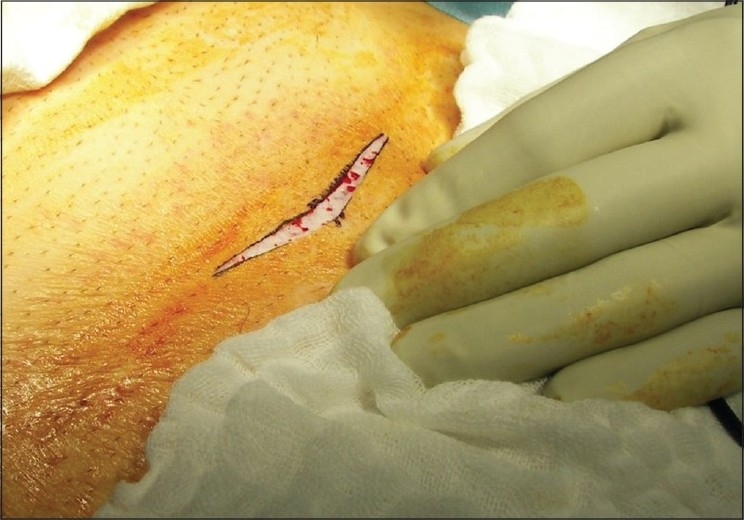
Transverse mini-incision

The transverse incision used was a modification of the Pfannenstiel incision that has been described by Manoharan *et al*.[4] Briefly, a 7-cm transverse incision is made in the lower abdomen, approximately 1-2 cm above the pubic symphysis [Figure 1]. Once the skin incision was made, the anterior rectus sheath was opened transversely, and the inferior rectus fascial flap was incised longitudinally down to the level of the pubic symphysis. The remainder of the operation proceeded in standard fashion.

**Figure 2:**
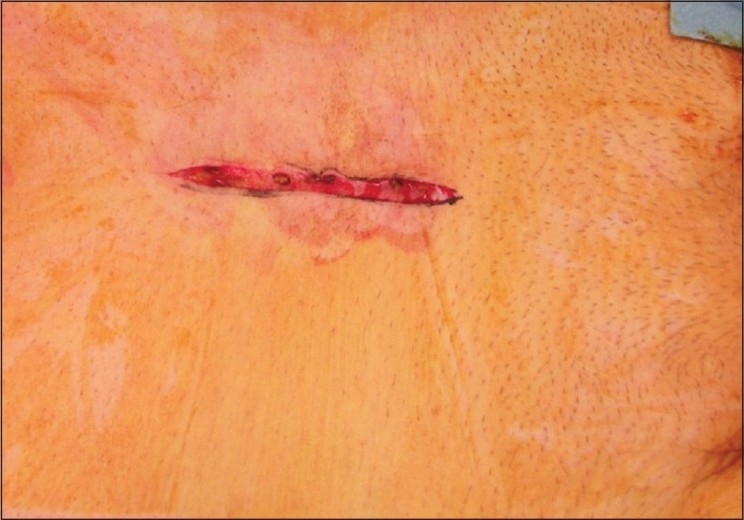
Longitudinal mini-incision

For patients randomized to a longitudinal incision, a 7-cm incision was made in the manner described by Marshall *et al*.[2] [Figure 2]. The rectus sheath was opened along the midline, the rectus muscles were separated, and the transversalis fascia was then opened. A standard Bookwalter retractor system[7] was used for all of the procedures.

The skin was closed with a subcuticular absorbable monofilament, and the wound was then covered with a dressing that concealed the incision. The dressing was left in place for at least 24 h in order to blind the patient to the method of incision. This is a technique suggested by Reidel *et al*.[8]

Upon completion of the surgery, the attending surgeon completed a self-administered survey which graded the technical components associated with each procedural step of the ORP using a visual analogue score (VAS). Duration of surgery, complications, and EBL were prospectively recorded. Cell saver is routinely available for all radical prostatectomies performed at our center. Postoperatively, all patients were placed on a patient controlled anesthesia (PCA) pump with morphine sulfate. Patients were instructed to resume a regular diet and were encouraged to ambulate independently within 24 h of surgery. On the morning after surgery, patients were started on oral oxycodone. One or two 5-mg tablets were administered every 4 h, as requested by the patient. Parenteral narcotics were given as needed, for breakthrough pain.

Postoperative pain and analgesic requirements were assessed at 4 h following surgery, and then every 12 h until discharge. Pain was graded with a self-administered VAS, and analgesic requirements were reported using morphine equivalents (ME). The LOS was defined as the number of hours that elapsed between the time that the dressing was applied and the time that the patient was discharged. Upon discharge, all patients were provided with 30 oxycodone 5 mg tablets, to be used every 4 h as needed. The catheter was removed at the first postoperative visit, between 7-10 days following surgery.

During the first postoperative visit, the Patient and Observer Scar Assessment Scale (POSAS) was administered to all patients. This is a validated scar assessment instrument that is used to assess wound healing and cosmesis.[9,10,11] It comprises two distinct series of VAS instruments: one is administered to the patient and one to the healthcare provider. In addition, two supplemental questions were added to assess the patient’s satisfaction with the appearance of the wound and the impact the incision had on his life. The responses to these two questions were structured as a VAS.

All data was prospectively collected and entered into a database. Statistical analysis was performed using SPSS 16. Chi- square and Student’s t- test was used to compare categorical and continuous variables, respectively.

## RESULTS

Sixty patients were randomized. Four of the 60 patients ultimately did not receive surgery. Two of them opted for radiation therapy, one failed medical clearance, and one declined participation following randomization. The remaining 56 patients underwent either a mini-transverse (n=27) or mini-longitudinal (n=29) incision ORP. Table 1 demonstrates that each group was balanced with respect to age, body mass index (BMI), prostate specific antigen (PSA), clinical stage, and pretreatment Gleason score. Additionally, technical difficulty, EBL, duration of surgery, postoperative pain scores, analgesic use, and LOS did not differ between the two incisions [Table 1]. None of the patients required extension of the incision in order to provide better exposure while working on the dorsal venous complex (DVC) and during vesico-urethral anastomosis. None of the patients required transfusion of allogenic blood. A transverse incision was associated with lower pain scores at the first postoperative visit, but this did not reach statistical significance.

**Table 1:** Comparison of select perioperative and early postoperative parameters

Parameter	Transverse	Longitudinal	*P*
N=56	27	29	
Age	59.8 ± 5.7	58.6 ± 3.8	0.455
BMI	28.338	27.981	0.723
PSA (mean)	7.46 ± 4.0	7.16 ± 4.07	0.783
T-Stage			
T1c/T2a	24	25	
T2b/c	3	4	
Gleason Score			0.979
5,6	15	17	
7	10	7	
>7	2	5	
Duration of surgery (min)	193 ± 41	186 ± 58	0.577
EBL (ml)	588 ± 311	812 ± 648	0.116
Allogenic PRBC Transfusion	0	0	1
Global difficulty (mean ± SD)	2.88 ± 2.5	3.30 ± 2.4	0.544
Postoperative pain score			
Immediate (4 h post op)	3.85 ± 2.62	3.88 ± 2.21	0.967
Day number 1 (AM)	2.81 ± 2.12	3.04 ± 1.61	0.658
Day number 1 (PM)	2.36 ± 2.02	2.67 ± 2.13	0.597
Day number 2 (AM)	2.10 ± 2.43	2.63 ± 1.97	0.433
Total inpatient analgesic requirements (MEs)	49.32 ± 49.6	68.81 ± 70.6	0.299
LOS (hours)	52.07 ± 22.9	61.04 ± 18.2	0.123
Mean VAS pain score at return visit	0.33	1.24	0.069
Mean number of oxycodone 5 mg tablets taken following discharge	13.29	10.33	0.4

There was one intraoperative rectal injury in a patient undergoing a mini-transverse incision ORP. This was repaired primarily, and did not result in any long-term consequences, other than increasing the LOS for this patient. There were only two minor postoperative complications (3.6%). One patient developed gross hematuria requiring an emergency room visit, and the other developed clostridium difficile enterocolitis. None of the patients who underwent mini-longitudinal incision ORP developed incisional hernia.

Individual mean responses to the POSAS showed excellent cosmetic success with both incisions. These are compared in Table 2. Overall, 98% of patients reported that the incision did not alter their appearance, and 96% reported that it did not change their life at all. Table 3 compares the pathological results post surgery between the two study groups. There were no significant differences noted between either groups in terms of extracapsular extension or positive surgical margins.

**Table 2:** Comparison of patient and observer scar assessment scale (POSAS)

	Transverse	Longitudinal	*P*
Patient scar assessment	Mean ± SD	Mean ± SD	
Itching	1.5 ± 0.9*	2.4 ± 2.3*	0.09
Color	2.13 ± 1.7*	1.48 ± 1.0*	0.11
Stiffness	2.33 ± 1.9*	2.15 ± 2.2*	0.76
Thickness	2.17 ± 1.6*	1.96 ± 2.0*	0.69
Irregular	1.46 ± 0.9*	1.46 ± 1.1*	1
Observer scar assessment	Mean ± SD	Mean ± SD	
Vascularization	1.63 ± 0.7*	1.33 ± 0.7*	0.42
Pigmentation	2.0 ± 1.1*	1.7 ± 1.1*	0.62
Thickness	1.75 ± 1.03*	1.71 ± 1.1*	0.95
Relief	1.63 ± 0.74*	1.57 ± 0.79*	0.89
Pliability	1.88 ± 1.0*	1.57 ± 0.79*	0.53

* For the subscales, a visual analog scale was used, which is divided between 1 to 10. The closer the response is to 1, the closer the response parallels normal skin.

**Table 3:** Comparison of post-surgery pathological results

	Transverse	Longitudinal	*P*
Number of Patients	n=27	n=29	
Pathological stage			0.159
p T2	21	23	
p T3/ T4	6	6	
Gleason Score			0.63
5-6	13	14	
7	9	11	
>8	5	4	
Margins			0.83
Negative	18	23	
Focal positive	5	2	
Positive	4	4	

## DISCUSSION

Anatomic ORP and pelvic lymphadenectomy has traditionally been accomplished using a midline vertical laparotomy. The optimal surgical incision provides the necessary exposure to permit surgery to be performed in a safe and efficient fashion. Limiting the length of the incision, splitting rather than transecting muscles, cutting within the direction of Langer’s lines, and utilizing natural skin folds in order to preserve cosmesis are well-established surgical principles. Abiding by these principles, perioperative morbidity can be minimized, convalescence can be facilitated, and cosmesis and overall patient satisfaction can be optimized.

The midline minilaparotomy radical prostatectomy incision was initially described by Marshall *et al*.[2] They demonstrated that an ORP and pelvic lymphadenectomy could be performed safely with a 7-8 cm lower midline incision, using a customized retractor system. ORP through Pfannenstiel incision has been described, and Salonia *et al*.[6] confirmed that it is equally safe and effective as the standard midline incision for performing ORP. The popularity of the lower transverse Pfannenstiel incision in gynecologic surgery arises from the fact that these incisions can easily be concealed, contributing to the aesthetic appeal of this approach. There have been no studies addressing cosmesis associated with a Pfannenstiel incision in men. We have adopted the use of a modified Pfannenstiel incision as the standard incision for performing ORP at our center.[4]

In this study, we have compared the use of two 7-cm mini-incisions that performed equally well for ORP. Patient demographics and preoperative clinical parameters were similar for both groups. No additional equipment, other than a standard Bookwalter retractor was necessary for either incision. There was no need for extension of the incision in any patient. Both incisions provided equally good exposure, and enabled the patients to have surgery with exceptionally low perioperative morbidity. The EBL was not significantly different among the study groups and there were no allogenic blood transfusions in either group.

As seen in Table 1, both the incisions that we evaluated were associated with low perioperative VAS pain scores. The 4-h postoperative pain score was almost similar in both groups, and subsequent measurements on post-op Day one and two were slightly lower in the transverse incision group. These differences however, did not reach statistical significance. Similarly, there were trends towards lower total analgesic requirements and a shorter LOS in the transverse incision group. Finally, the mean VAS pain score at the return visit to clinic was 0.33 in the transverse group and 1.24 in the longitudinal incision group (*P*=0.06).

Aside from the functional aspects of the two mini-incisions that we studied, one unique aspect of our study is the incorporation of an instrument that assesses the cosmetic aspects of the surgical wound. Scars are an inevitable result of surgery that may have functional, cosmetic, and psychological consequences for the patient.[10] The impact of the surgical scar in men undergoing prostate cancer surgery has not been previously studied. The POSAS is a scar assessment tool that has been validated for the assessment of linear surgical scars.[10,11] It comprises two distinct questionnaires which are independently completed by both the observer, and the patient. The fact that the patients express their own opinion concerning the appearance of the scar represents a distinct advantage of the POSAS over other scar assessment instruments, such as the Vancouver Scar Scale.[12] An additional advantage of the POSAS is that it provides an objective measure of itching and pain, which have been found to weigh heavily in a patient’s general opinion of a wound,[9,11] especially in the early postoperative period. Color, stiffness, thickness, and irregularity of the scar are additional parameters that are uniquely assessed by the POSAS, and seem to have the greatest bearing on a patient’s long-term cosmetic impression of a surgical scar.[11]

Our study indicates that both, the longitudinal and transverse 7-cm incisions provide equally high levels of cosmetic satisfaction for the patients, at least within the early postoperative period. None of the individual parameters within the various subscales were found to be different between the two incisions, and the overall scoring indicated little difference in the wound from normal skin. Additionally, the responses to our global supplemental questions confirmed that neither of the two mini-incisions had a significant impact upon the men’s self-image, or their life in general. Follow-up will be important in order to determine whether changes within the maturing surgical wound influence the individual patient’s attitude or satisfaction with the scar. From an oncologic standpoint, our study demonstrated that neither of these two different types of incisions made any significant difference in the clinicopathologic outcomes. However, follow-up is important to study the effect on long-term oncological efficiency.

While this data is encouraging, it should be noted that our study is not without limitations. Postoperative pain and analgesic requirements are difficult to compare between patients undergoing surgery at different institutions, even when VAS measures are utilized. Wu *et al*.,[13] showed that there may be wide temporal variations of the VAS score in the perioperative period, which depend upon whether the patient is engaged in activity or rest during the assessment. Also confounding the interpretation of VAS scores is the fact that patients may use higher quantities of analgesics, which may paradoxically mask higher levels of pain that they are experiencing.[14]

It may be that the preoperative cosmetic attitudes of men undergoing prostate cancer surgery are superseded in importance by other functional aspects of surgery such as sexual function and urinary continence. Alternatively, cosmesis associated with a lower abdominal incision in a hair-bearing area may not pose a concern for men. However, this argument would not hold in this robot-assisted minimally invasive surgical era, where patients seek excellent cosmesis with exceptionally low morbidity. The authors strongly believe that the 7-cm mini incision ORP would offer an option for centers which have not ventured into the newly available ultra-expensive surgical facilities. The technique can be accomplished with little additional training for the urologist who is experienced at ORP, and with no additional costs. Exploration of these issues in other groups of men, particularly those seeking minimally invasive prostate cancer surgery is warranted in the future.

## CONCLUSIONS

We have evaluated the feasibility, safety and a number of perioperative parameters associated with performance of ORP through a 7-cm transverse or longitudinal incision. Both incisions provide adequate exposure for the safe performance of ORP. In addition, both these incisions are associated with low postoperative pain and analgesic requirements, allow for early convalescence, and a short postoperative LOS. They both provide highly satisfactory cosmetic results for the patient.
